# Assessing the accuracy of blood RNA profiles to identify patients with post-concussion syndrome: A pilot study in a military patient population

**DOI:** 10.1371/journal.pone.0183113

**Published:** 2017-09-01

**Authors:** Jimmaline J. Hardy, Scott. R. Mooney, Andrea. N. Pearson, Dawn McGuire, Daniel. J. Correa, Roger P. Simon, Robert Meller

**Affiliations:** 1 Neuroscience Institute, Morehouse School of Medicine, Atlanta, Georgia, United States of America; 2 Neuroscience & Rehabilitation Center, Dwight D. Eisenhower Army Medical Center, Fort Gordon, Georgia, United States of America; 3 Department of Medicine, Morehouse School of Medicine, Atlanta, Georgia, United States of America; Cleveland Clinic, UNITED STATES

## Abstract

Mild traumatic brain injury (mTBI) is a complex, neurophysiological condition that can have detrimental outcomes. Yet, to date, no objective method of diagnosis exists. Physical damage to the blood-brain-barrier and normal waste clearance via the lymphatic system may enable the detection of biomarkers of mTBI in peripheral circulation. Here we evaluate the accuracy of whole transcriptome analysis of blood to predict the clinical diagnosis of post-concussion syndrome (PCS) in a military cohort. Sixty patients with clinically diagnosed chronic concussion and controls (no history of concussion) were recruited (retrospective study design). Male patients (46) were split into a training set comprised of 20 long-term concussed (> 6 months and symptomatic) and 12 controls (no documented history of concussion). Models were validated in a testing set (control = 9, concussed = 5). RNA_Seq libraries were prepared from whole blood samples for sequencing using a SOLiD5500XL sequencer and aligned to hg19 reference genome. Patterns of differential exon expression were used for diagnostic modeling using support vector machine classification, and then validated in a second patient cohort. The accuracy of RNA profiles to predict the clinical diagnosis of post-concussion syndrome patients from controls was 86% (sensitivity 80%; specificity 89%). In addition, RNA profiles reveal duration of concussion. This pilot study shows the potential utility of whole transcriptome analysis to establish the clinical diagnosis of chronic concussion syndrome.

## Introduction

Concussion, or mild traumatic brain injury (mTBI), is a common injury sustained in both military and civilian populations. At present, there are no pathognomonic signs of concussion in the absence of loss of consciousness, which may not be a reliable indicator of severity[1, 2]. In prospective studies, when followed for 6- to 12-months, a minority of concussion cases will go on to experience a poor clinical outcome associated with persistent multi-symptom complaints (≤6% of cases) or inability to return to work/normal activities (≤ 4% case):[3] post-concussion syndrome (PCS). PCS can be challenging to diagnose. Concussion diagnosis relies heavily on subjective self-report of non-specific symptoms that may or may not be medically verifiable via medical records, physical/neurological examination, neuroimaging, and/or neurocognitive testing.[4] Furthermore chronic concussions have been associated with heightened risk of neurodegenerative conditions.[5] As such, a sensitive, objective test for identifying potential patients with post-concussion syndrome is urgently needed.

Here, we propose that blood transcriptome profiling may hold promise as a method of identifying potential biomarkers for diagnosing chronic concussion. This approach is based on the premise that circulating blood cells act as sentinels for the whole body. Different disorders or damage to the CNS may impart differential effects on the transcriptome in these cells[6]. Such an approach shows remarkable sensitivity and specificity for stroke diagnosis and prognosis [7–9]. Recent studies have identified differentially expressed genes in blood following acute concussion, acute sports related concussion, and blast-TBI via microarray technology, [10, 11] however prediction modeling was not performed. This approach also holds some advantages over single (protein) biomarkers because the patterns of gene expression can encode more clinically-relevant information than the diagnosis alone. We hypothesize that blood transcriptome analysis is sensitive and specific enough for use as an objective biomarker for the diagnosis of post-concussion syndrome.

## Results

### Patient samples

**Fig 1:**
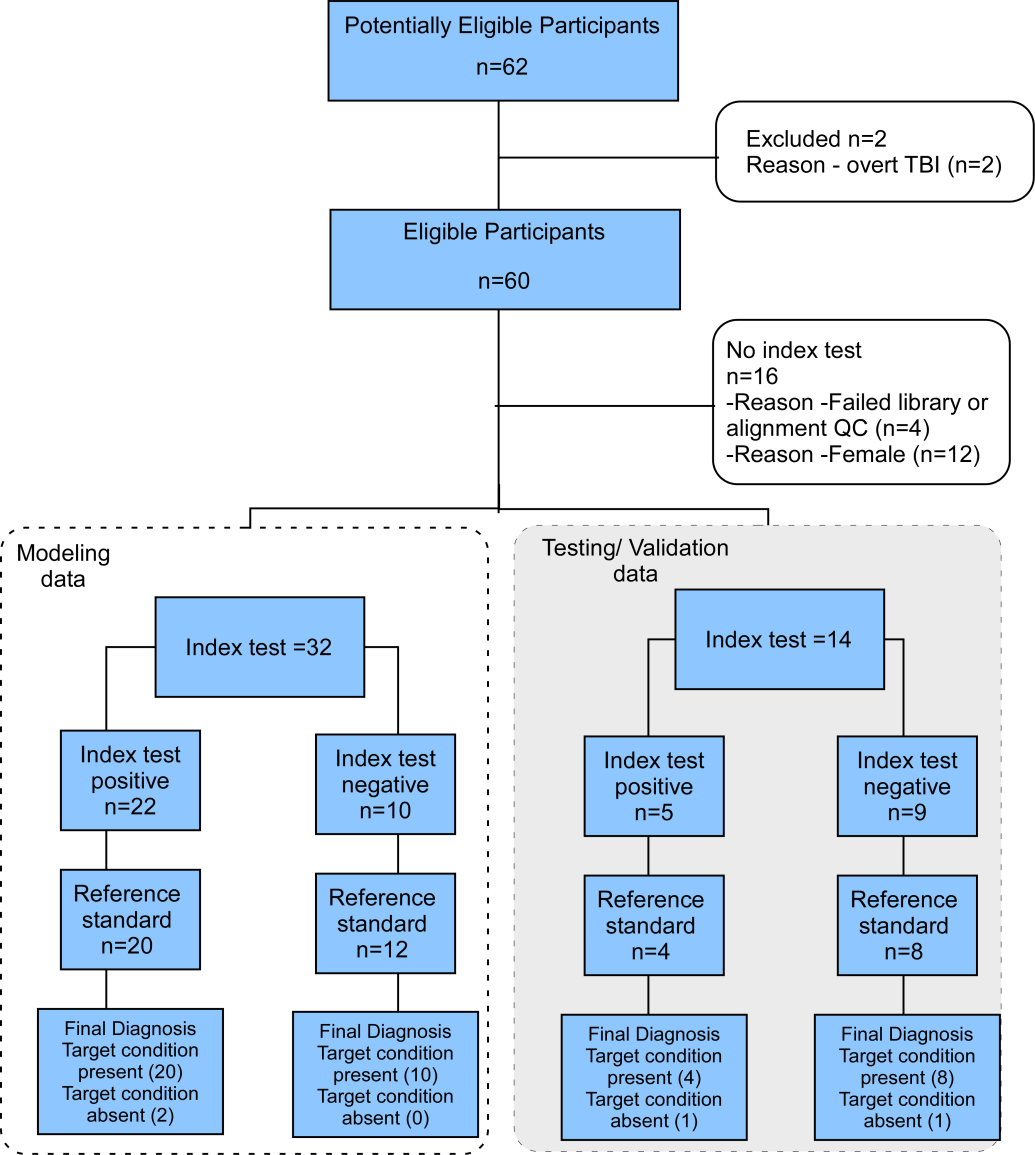
Flow chart of study. Potential participants (62) were recruited from the Neuroscience and Rehabilitation Center–TBI Clinic at the Dwight D. Eisenhower Army Medical Hospital, Fort Gordon, Georgia. Two were found to be ineligible for the study due to moderate or severe TBI/tumor upon further review of medical chart. From the remaining 60 patients, we excluded 4 samples due to failed library QC, low RNA yield or abnormal mapping. Females were ultimately excluded from the study due to low recruitment (n = 11; one with failed library QC). Subjects (n = 46) were split into a modeling set and a second validation set. The modeling set was used to create an algorithm (Index test) for determining the clinical diagnosis of post-concussion syndrome (positive outcome). This was compared to the confirmation of cognitive symptoms consistent with post-concussion sequelae (Reference test). The algorithm was tested on the modeling data set (n = 32) and the test data set (n = 14). Performance on the modeling data was 94% accuracy, and 86% accuracy in the independent validation set.

Sixty active duty Service Members (SMs) with and without a history of remote (non-acute; i.e., > 90 days post injury) clinically diagnosed concussion were recruited (Fig 1) from a military Traumatic Brain Injury Clinic. Verification of the diagnosis of chronic concussion (post-concussive syndrome) was made by a board certified neuropsychologist following review of medical documentation in accordance with DoD/VA TBI CPG criteria.[12] Following extraction of RNA and sequencing, four samples were excluded; due to an RNA yield less than 500 ng (1 sample), less than 5 million alignments of cDNA to the hg19 reference genome (2 samples), or abnormal mapping statistics (1 samples) (Fig 1). Due to a low enrollment of females (11 out of 60) we focused our analysis on the male subjects (49). The modeling subset comprised of twenty patients with chronic concussion and twelve controls without a history of concussion (Table 1). A second, testing set was assembled and consisted of 14 male samples comprised of five chronic concussion and nine controls. There was no statistically significant difference in RNA-yield (1920 vs. 2393 ng p = 0.381: unpaired t-test) between the modeling and testing data sets.

**Table 1:** Demographics of males subjects in the study.

	Control	Concussion	Significance
Diagnosis	21	27	ns
Age	37.0 ±8.7	31.5 ± 7.0	0.0211
Ethnicity			ns
*African American*	2	7	
*Asian/Pacific Islander*	1	0	
*Caucasian*	15	18	
*Hispanic*	3	2	
Number of concussions	n/a		n/a
*1–3*		5	
*4–6*		12	
*7–9*		4	
*>10*		6	
Time since last concussion*	n/a		n/a
*6–12*		4	
*13–24*		8	
*>25*		14	
BMI*			0.0103
*healthy*	7	1	
*overweight*	9	13	
*obese*	4	13	
Blood Pressure			0.0071
normotensive	5	14	
prehypertensive	11	3	
hypertensive	5	10	
PTSD	2	18	<0.0001
Depression	2	12	0.0108
Alcohol/Drug	0	3	ns
Antidepressants	2	14	0.0023
Anti-anxiety	1	14	0.0005

### Alignment and mapping

**Fig 2:**
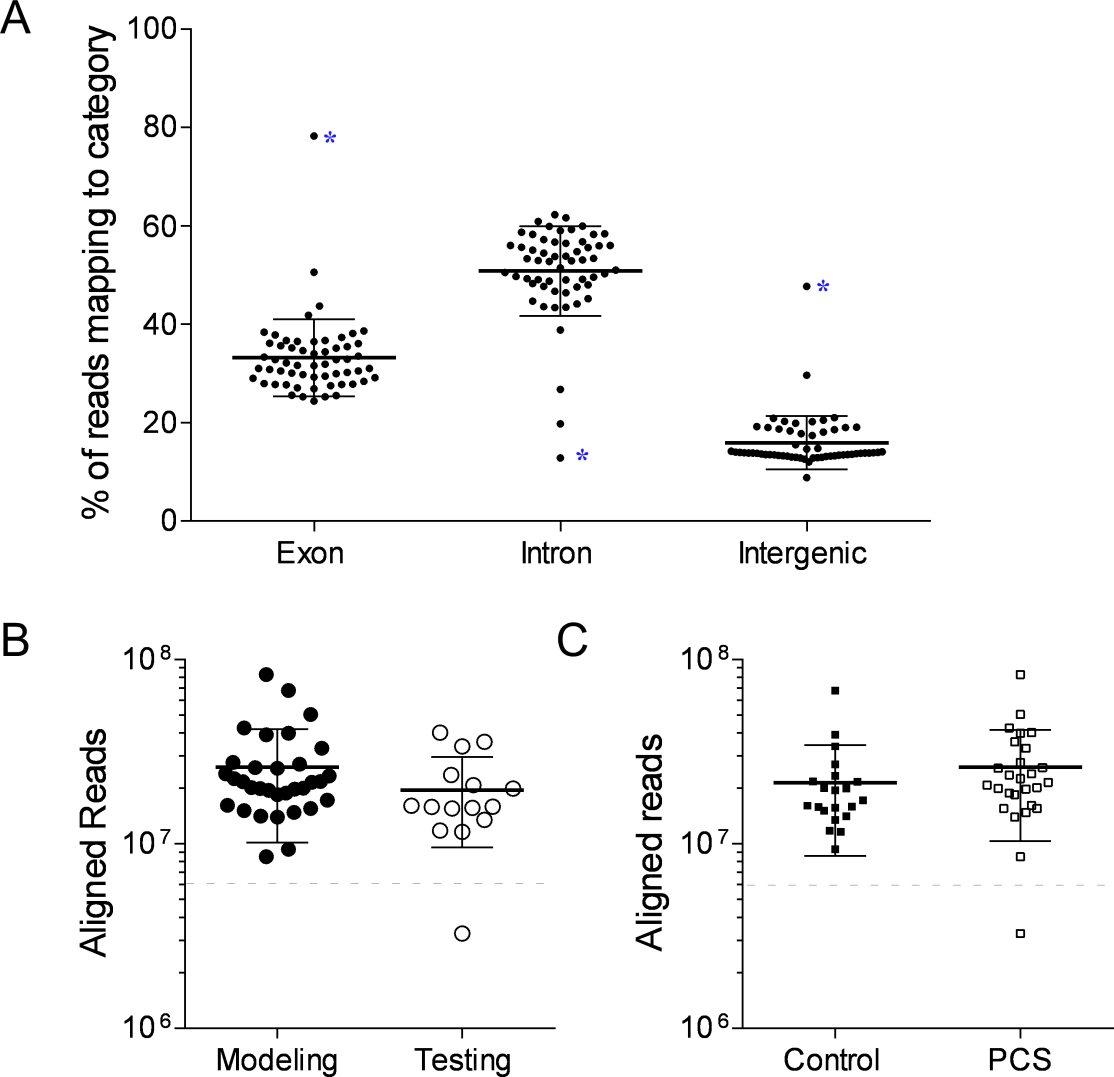
Male gene read alignment to the hg19 reference genome and mapping to the RefSeq Annotation Guide. A. Average mapping proportions were 33.2% exonic, 50.8% intronic, and 15.9% intergenic. Blue asterisk indicates excluded sample. B & C Average alignment was 24 million reads/ sample. Data are shown stratified by clinical condition (B: PCS vs Control) and data group (C: Modeling vs Testing). The dashed line denotes the 5 million read cut off.

The average number of alignments to the human hg19 reference genome for male samples (n = 46; model = 32; test = 14) was 24.0 million ± 15.9 million. Mapping of RNA to the RefSeq Transcripts annotation guide revealed 33.2% of the reads aligned to exons (i.e. RNAs encoding proteins, microRNAs and known non-coding RNAs), 50.8% to introns and 15.9% intergenic (between known genes) (Fig 2A). There were no significant differences in the number of alignments/ sample between the modeling and testing data sets (p = 0.1555, unpaired Student’s t-test), or between the control and chronic concussion data sets (p = 0.2890: unpaired Student’s t-test) (Fig 2B and 2C).

**Fig 3:**
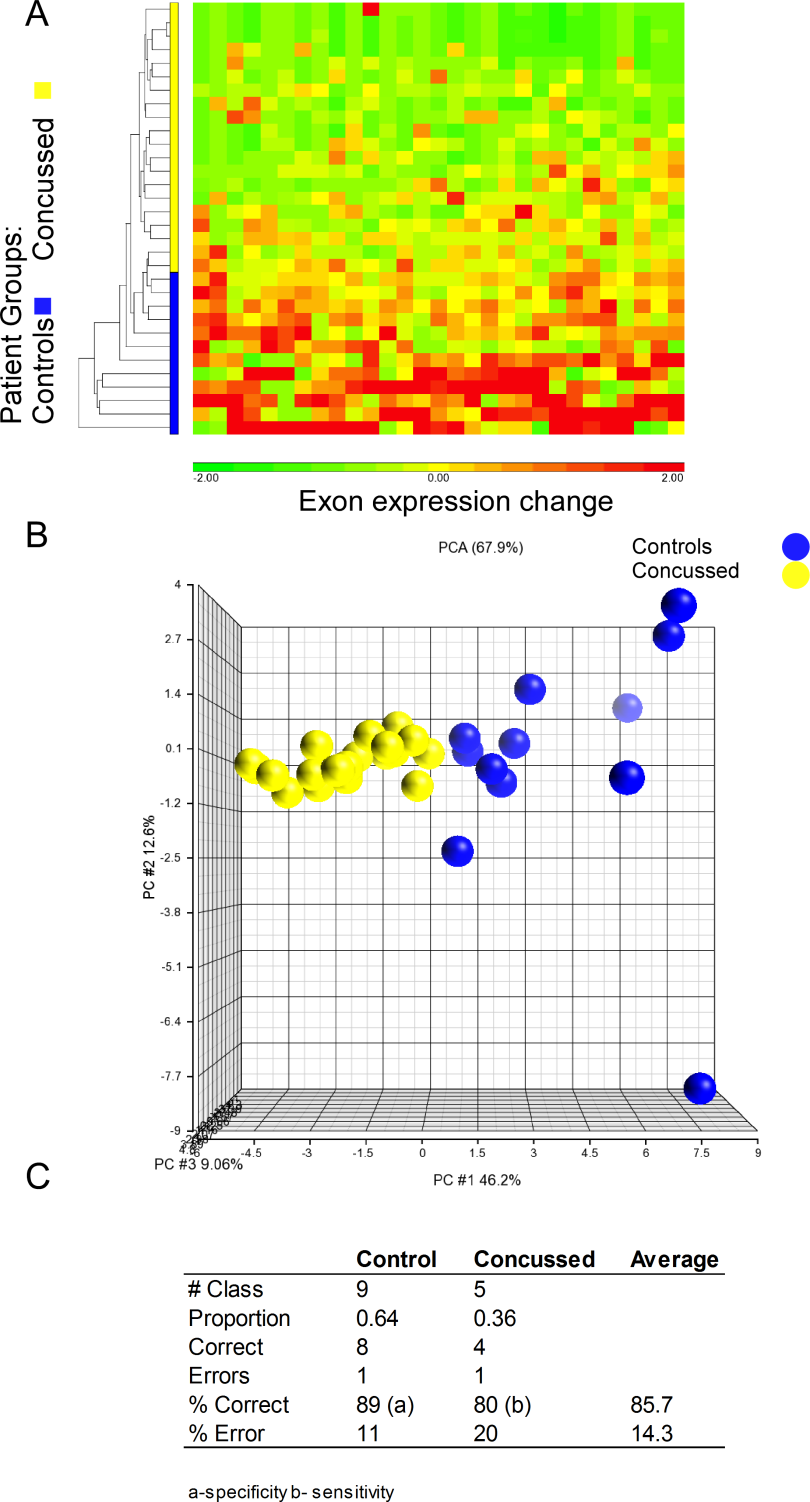
Exon expression pattern of concussion predicts clinical diagnosis. A. Hierarchical clustering shows pattern of separation between concussed and controls based on expression of 29 exons (x-axis) B. Principle component analysis plot shows the exon expression patterns separate due to concussion status. C Results of testing SVM generated model of 25 exons (Table 2) on testing data set, using a diagnosis of PCS/ concussion as the positive call. Note the accuracy was 86%, sensitivity 80% and specificity of 89%.

Based on our previous experiences, we focused our study on exon expression values, rather than whole gene expression values.[9] The exon expression data were filtered to remove low expressed RNAs and normalized for library size (reads per kilobase per million mapped (RPKM)). The data were further normalized to the trimmed mean value of the filtered data set. The resulting data were subjected to one-way ANOVA using PCS diagnosis as the independent variable. To perform cluster analysis and prediction modeling, we relaxed our p-value to an unadjusted p < 0.001, yielding 29 differentially expressed exon values, with an overall fold change that ranged from -10 to -2.1 (parameters based on [9]). Interestingly, exon expression levels were predominantly decreased in the PCS group compared to controls (Fig 3A). Hierarchical clustering of these exon expression values resulted in a separation of PCS patients from controls (Fig 3A). Principal component analysis (PCA) of the regulated exons show a separation between patients with PCS and controls. Three primary components accounted for 67.9% of the total variance. (Fig 3B).

### Exon expression and post-concussion syndrome diagnosis

We tested the ability of modeling algorithms to correctly classify patients based on their known diagnosis of PCS using exon expression patterns. The list of expression values from the 29 differentially expressed exons (above) were investigated using support vector machine modeling with shrinking centroid variable selection and sigmoidal kernelling. We specifically identified the most accurate models in training (>85% AUC) with the fewest number of exon variables based on two level and bootstrap cross validation of the models with the training set.

The best prediction model developed with the training group (94% accurate (equivalent to Area Under the Curve (AUC)), sensitivity 100%, specificity 83%), was then assessed using the 14 test samples that were filtered and normalized as above (Table 2). The model correctly identified 86% of patients with their clinical condition (PCS or control) (12 out of 14) with a sensitivity of 80% (4 of 5; 95% confidence interval 51–99%), specificity of 88% (8 of 9; 95% confidence interval 28–99%)), and AUC of 84% (SVM model, sigmoidal kernelling, 25 exon variables, cost 201, nu 0.5, gamma 0.0001, tol 0.001: positive outcome = concussion) (Fig 3C: Table 2). The results suggest a positive association of our test and the correct clinical diagnosis (OR = 32: 95% CI 1.5–656; p = 0.023).

**Table 2:** Exon expression values that give most accurate prediction of chronic concussion in testing data set. Note exon values give chromosomal location, based on RefSeq annotation guide.

Model	Shrinking Centroids, SVM c_svc, cost 201, nu 0.5, tol 0.001, kern sigmoid, deg 3, gamma 0.0001, coef0 1
Variable Name	
chr1.1330774–1330895>CCNL2	
chr1.220279233–220279403>IARS2	
chr1.247019032–247019130>AHCTF1	
chr11.117067946–117068121>LOC100652768	
chr12.10532299–10532392>KLRC4-KLRK1	
chr12.50867198–50867320>LARP4	
chr13.28008276–28008357>GTF3A	
chr15.67879184–67879252>MAP2K5	
chr16.47536902–47537002>PHKB	
chr17.1582585–1582705>PRPF8	
chr17.67410838–67411142>MAP2K6	
chr17.74483794–74483992>RHBDF2	
chr19.39879200–39879317>PAF1	
chr2.122273241–122273339>CLASP1	
chr20.40122178–40122313>CHD6	
chr3.129017197–129017379>HMCES	
chr4.190873317–190873443>FRG1	
chr5.118510956–118511033>DMXL1	
chr6.26406137–26406485>BTN3A1	
chr6.88315635–88315740>ORC3	
chr7.17838632–17838778>SNX13	
chr7.48018014–48018191>HUS1	
chr7.50459427–50459562>IKZF1	
chr8.141310568–141310714>TRAPPC9	
chrX.71710779-71710857>HDAC8	

### Expression patterns associated with duration since last concussion

**Fig 4:**
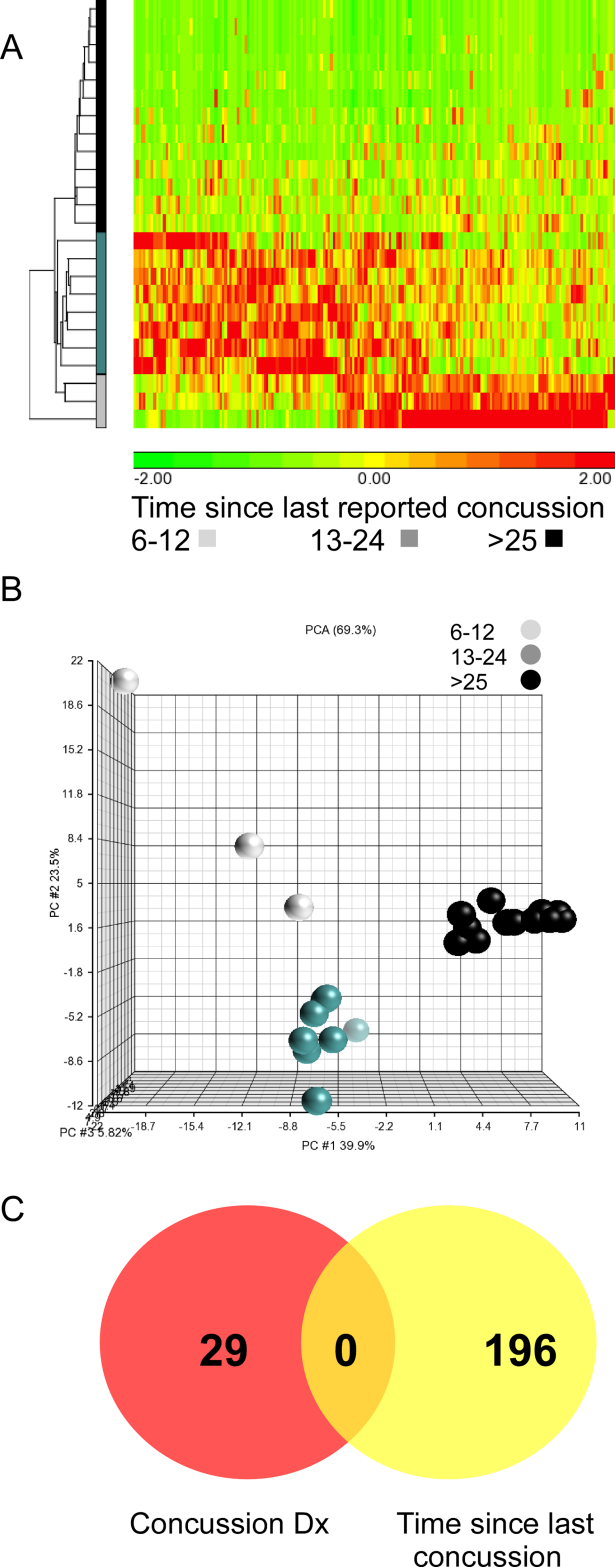
Exon expression pattern of concussion duration. A. Hierarchical clustering of patient’s time since last concussion based on 196 exons. B. Principle component analysis plot shows separation of data based on duration since last concussion. C. Venn diagram shows no overlap of regulated exons between concussion diagnosis and time since last injury.

To determine whether additional medically useful information could be encoded within the transcriptome, we investigated changes in exon expression profiles that correlate to the reported duration since the last concussion (stratified as: <12months, 13–24 months and > 25 months). PCS patients from both training set and validation set were combined, and subjected to one-way ANOVA with time since last concussion as the variable. We relaxed our p-value to an unadjusted p < 0.001, yielding 196 differentially expressed exon values (six pass an FDR of 0.05). Hierarchical clustering and PCA (69.3%) showed a clear separation between the data groups illustrating the potential of unique patterns of exon expression for stratifying concussion based on time since last incident (Fig 4A and 4B). Of note, there was no overlap between exons that were used in the prediction modeling for PCS diagnosis and the exons identified as being associated with duration since last concussion (Fig 4C). We were unable, however, to perform prediction modeling due to low sample size in each individual group.

## Discussion

The goal of this study was to assess the accuracy of predicting the diagnosis of post-concussion syndrome (PCS) in a military cohort using blood RNA expression profiling. The test was developed in a subset of recruited participants, and then validated in a separate testing set of participants. We show profiles of RNA expression in peripheral blood have an 86% accuracy rate at correctly stratifying patients with PCS. We also show a persistence of RNA expression profiles following concussion. To our knowledge, this is the first study to demonstrate the predicted efficacy of complete transcriptome analysis via RNA sequencing at discriminating the diagnosis of persistent post-concussive syndrome from controls.

The diagnosis of chronic concussion is challenging, and while consensus statements describe symptoms of concussion and PCS, [13] the exact determination is still more often than not reliant on subjective report by the patient in the absence of medical/emergency medical service, combat medic, or other clinical provider documentation close in time to the alleged event(s). Acute concussion can be determined using computerized neuropsychological testing. The accuracy rates we observe in our pilot study are comparable to the Immediate Post Concussion assessment and Cognitive Testing (ImPACT) test (sensitivity = 81.9%, specificity = 89.4%), a previously validated computer based neurocognitive screening tool commonly employed in concussion evaluation.[13] However, ImPACT is predominantly used in an acute setting, and our patient population suffers from chronic symptoms (>6 months). An objective blood based biomarker may be more rapid and less costly than neuropsychological evaluation that are used to help appreciate cognitive and behavioral deficits that would be consistent with or supportive of a diagnosis of PCS.

Our data are consistent with previous blood gene expression studies of concussion, which show a predominant downregulation of gene expression following sports concussion,[11, 14] as well as military personnel with chronic symptoms of mild traumatic brain injury. [10] There are major differences between our study and those. First technologically, we use RNA-Seq to identify RNA in our samples, not microarray (see [15, 16]). Second, previous studies investigate whole gene expression values, where as in this study we use exon expression profiles. Our previous study suggests exon expression values to have greater diagnostic utility.[9] We use whole peripheral blood as a simple biofluid for biomarker assessment, because peripheral blood mononuclear cell isolation procedures affect transcription.[17] Finally, while previous studies focused on potential biological mechanisms (NFKB and growth factor regulation,[10, 14]) we focused on the accuracy of this approach for clinical diagnostic purposes. It should be noted that pathway analysis tools are not designed to be used with exon profile data. This is especially pertinent as it is unclear how long term changes in blood transcription are affected by concussion/mTBI events. Finally, it is also interesting to contrast transcriptome effect following mTBI [10, 11, 14]with that of other neurological conditions. For example, brain injury sustained following stroke increases gene expression in blood.[6, 9] While there are clear differences with respect to time of blood withdrawal from injury, this does suggest a clear difference in transcriptomic response to different injuries, and was previously shown with different animal models of brain injury.[18]

With regards to duration since the last concussion, we observe a clear stratification of data. There is no overlap of exon values differentially expressed with respect to duration since last concussion and the diagnosis of concussion as used for modeling. In addition, we saw little overlap between exon expression profiles between PCS and other co-morbidities, blood pressure or body weight in our patients (one exon in common, RM: Personal Communication). These observations suggest that additional clinically relevant information may be encoded by unique transcriptome responses following injury which may have utility for diagnosis. Further characterization of longitudinal RNA signatures during the evolution and resolution of chronic concussion may enable the development of markers of recovery or for the assessment of therapeutic interventions.

### Study limitations

The clearest limitation of this study is the sample size. This was a pilot study to investigate the feasibility of this approach, and as such requires expanding to a larger cohort. We recruited samples for modeling and verification from the same center, albeit at different times. Since concussed patients were self-determined, this could represent a potential source of bias. Clearly this may be resolved by a larger multi-site trial. An additional confounding factor is that concussion is a diagnosis based on evidence and symptoms. Because of sample size we were unable to fully evaluate a differentiation of each of the subjective symptom components of PCS (depression, affect changes, dizziness, fatigue, sleep problems, concentration/memory performance, apathy or personality changes). In the absence of objective diagnostic markers, however, the ability of RNA-seq to diagnose concussion is reliant on the accuracy of the initial clinical diagnosis. To this end, the patients were clinically confirmed as manifesting PCS after review of available medical documentation, review of alleged concussive event(s) in terms of peri-event details and symptoms in accordance with DoD/VA mTBI CPG criteria, within the setting of credible cognitive deficits demonstrated on performance based, norm referenced neuropsychological testing. A further concern could be the underreporting of concussion in the control patient population, therefore additional control patients should be investigated in a larger study (mild orthopedic injury for example).

For this pilot investigation, we chose to focus on male patients to reduce the confounding factor of sex. Recent studies suggest gender differences in concussion severity and resolution in females compared to males, indicating that a sex specific biomarker panel may be required.^15^ In addition, we did not stratify our patients by race. Increased sample size will enable analysis, modeling, and prediction based on the interaction of race, sex, and other potential confounding factors, such as elapsed time and number of injuries, presence of comorbidities, and injury source (primary blast wave versus rotational or mechanical forces). However, the minimal overlap of differential RNA expression patterns for different concurrent co-morbidities suggests that different neurological conditions result in unique RNA expression profiles.

### Technical considerations

From a technical standpoint, the use of whole blood derived RNA with ribosomal RNA depletion, as opposed to peripheral blood mononuclear cell extracted RNA (PBMCs), could also account for dissimilarity of identified genes compared to other studies.^6^ Whole blood was used to simplify our library building procedure. In addition, this approach has been used for other biomarker studies.[6] It is not yet clear how peripheral blood RNA expression is associated with brain pathophysiological processes; however multiple studies show peripheral gene expression changes in response to brain injury, and that different forms or causes of injury have different profiles.[8–11, 19–21]

Sequencing of total RNA derived samples resulted in relatively equal mapping of reads to gene regions (1/3 exonic, 1/3 intronic, and 1/3 intergenic).^9^ Following ribosomal RNA depletion and annotation to a different reference guide (Aceview vs Refseq), we observed a ~42% exonic, 49% intronic, and 10% intergenic distribution. This suggests that ribosomal depletion and choice of alignment annotation alters the proportion of the sequenced transcriptome and comparison to whole transcriptome derived expression values may not be appropriate. Consequently, caution should also be taken when performing depletion strategies.

Following alignment of RNA derived cDNA libraries to the human genome, we annotated gene (exon) location using Ref-seq. Other annotation guides are available and may yield different mapping statistics. Approximately 13% of the known reads mapped between genes (intergenic). Two of the differentially expressed exons associated with persistent concussive symptoms were identified as uncharacterized, noncoding RNA. These novel regions may further aid in diagnosis of concussion. Indeed, our previous study utilized a novel annotation guide to create prediction models of stroke diagnosis and prognosis.^9^ Therefore, additional mechanisms and functions of differentially regulated non-coding (novel) RNAs may be observed as annotation guides become more comprehensive.

### Conclusion

The long-term goal of our research is to develop an easily deployed, reliable, sensitive clinical assay for rapid and objective detection of post-acute concussive symptoms including persisting cognitive sequelae in military personnel. The first step toward achieving this goal was our investigation into the utility of gene exon expression analysis. Using whole transcriptome analysis of peripheral blood, we have identified a signature exon panel capable of discriminating between post-concussion syndrome and non-concussed military controls. With additional research in a larger population and future validation there is potential for this novel approach as a method for diagnosis based on objective, quantifiable changes in the transcriptome. An objective biomarker test may minimize reliance on subjective self-report, and/or costly and less portable neurocognitive and neuroimaging procedures. This study also highlights the wealth of information than can be ascertained through precision medicine techniques, particularly RNA-Seq, that can assist with the diagnosis of complex disorders.

## Methods

### Study approval and informed consent

This study was approved by the Morehouse School of Medicine (MSM) and Dwight D. Eisenhower Army Medical Center (DDEAMC) Institutional Review Boards. Written, informed consent was obtained from all individuals prior to participation. The patient’s capacity to consent was based on evaluation by the clinical research coordinator (RN Nurse). This evaluation included assessment of ambulatory function, alertness and orientation, in addition to formal neuropsychological testing of neurocognitive function. Blood samples and study questionnaires were de-identified before analysis. All requests to access the raw sequencing data set from this study should be made to the Fort Gordon IRB (usarmy.gordon.medcom-eamc.mbx.irb@mail.mil).

### Patient recruitment

Between April 2014 and February 2015, 30 active duty Service Members (SMs) with history of remote (non-acute; i.e., > 90 days post injury) concussion who were manifesting symptoms (i.e., multiple post-concussive symptoms within the setting of credible objective evidence of cognitive dysfunction on performance based neurocognitive testing) were recruited from a DDEAMC TBI Clinic. SMs with a history of remote (non-acute; i.e., > 90 days post injury) clinically diagnosed concussion were recruited (Fig 1) from a military Traumatic Brain Injury Clinic. The diagnosis of chronic concussion (alternatively called chronic post-concussive syndrome) was made by a board certified neuropsychologist with > 8 years of post-doctoral experience working with military TBI patients after reviewing SMs available medical documentation (e.g., CONUS/OCONUS documentation available in the DoD/military global electronic medical record such as duration of altered mental status/LOC, presence of neurological soft signs, post-event symptom topography report(s), GCS scores, Military Acute Concussion Evaluation scores, neuroimaging, ANAM-IV), conducting a diagnostic clinical interview where alleged civilian and military related concussion history(ies) and peri-event sequelae were explored with subsequent staging of presumed TBIs in accordance with DoD/VA TBI CPG criteria—all within the setting of credible evidence of cognitive dysfunction as elucidated by norm-referenced, performance based, neurocognitive testing procedures (To include various combinations of: Automated Neuropsychological Assessment Metrics–IV TBI Modules, Booklet Category Test, Brief Visuospatial Memory Test-Revised, California Verbal Learning Test-II, Connor’s Continuous Performance Test-II, Digit Vigilance Test, Expanded Controlled Word association Test, Personality Assessment Inventory, Reliable Digit Span, Repeatable Battery for the Assessment of Neuropsychological Status, Test of Memory Malingering, Trail Making Tests, Wechsler Abbreviated Scales of Intelligence, Wide Range Achievement Test-Revised, Wisconsin Card Sorting Test). Study participants underwent a 6 ml blood draw (2 x 3ml), had vital signs recorded, and completed a demographics questionnaire regarding military, medical, and concussion histories (Table 1). Individuals were compensated $40 for their participation. Self-reporting of medical and concussive histories was verified by second author (SRM).

### Whole transcriptome assembly, sequencing, and alignment

Three ml of blood were collected in PAXgene™ vacutainer tubes (PreAnalytiX). Blood was frozen and transported on dry ice from DDEAMC to MSM where it remained frozen until RNA extraction. Total RNA was isolated using the Paxgene™ Blood RNA Kit 50, v2. Initial RNA quality was assessed via the Agilent RNA 6000 Nano Kit and 2100 Bioanalyzer.

Total RNA was subjected to ribosomal RNA (rRNA) depletion via a modified version of the Life Technologies Ribominus™ Eukaryotic System v2 protocol. In short, RNA was incubated in a 2.5% formamide (Sigma) solution for 2 minutes at 95°C prior to rRNA removal. Hybridization and separation of rRNA was carried out via the Ribominus™ Core Module v2 at one half the suggested volumes. rRNA depleted samples were purified with the Ribominus™ Concentration Module. rRNA reduction was verified with the Agilent RNA 6000 Pico Kit and 2100 Bioanalyzer.

Whole transcriptome cDNA libraries were constructed according to the Life Technologies low input protocol using the SOLiD Total RNA-Seq Kit (4445374) with the following modifications. Approximately 200 ng of rRNA depleted RNA were incubated with 3μl RNase III for 3 minutes at 37°C. Four μl digested RNA were hybridized with 1μl adapter and 3μl hybridization solution. cDNA was synthesized according to the manufacturer’s protocol. Two rounds of size selection were carried out with Agencourt Ampure XP beads at 1.8x then 1.6x concentrations. cDNA was then amplified with barcoded primers at initial denaturation of 95°C for 5 minutes followed by 24 cycles of 95°C for 30 seconds, 65°C for 30 seconds and 72°C for 30 seconds. Final extension setting was 72°C for 7 minutes. An additional 3 rounds of size selection were carried out with 1.6x Ampure XP beads. cDNA quality was assessed via the Agilent High Sensitivity DNA Kit and 2100 Bioanalyzer. cDNA was quantified via real time PCR on the Bio Rad C1000 Thermal Cycler with the TaqMan Universal PCR Master Mix and artificial primer (AC00010015_a1). Five hundred pM composites of 8 libraries each were amplified with emulsion PCR and enriched for monoclonal beads with a target deposition of 500 million beads per flow chip lane. Composite libraries were sequenced in triplicate (single end reads, 3 lanes per flow chip) in 40 or 45 base pair fragments on the SOLiD 5500xl analyzer. This workflow is available at https://protocols.io/view/blood-derived-cdna-library-construction-for-sequen-idgca3w

### Mapping, quantification, and differential expression

Data from the SOLiD sequencer (xsq format) of cDNA reads were aligned to the hg19 reference genome using LifeScope Genomic Analysis Software, and output aligned dat files (Bam format) were uploaded to a Dell workstation running Partek Genomics Suite. The RNA-seq workflow in Partek Genomics Suite was used to determine gene, transcript, and exon abundances based on the RefSeq annotation guide (02-02-2016). Raw reads were filtered to include those with values greater than 10 in 10% of the samples. The resultant list was used to filter the exon read per kilobase per millions mapped (RPKM) datasheet. RPKM values were further normalized by dividing by the trimmed mean (10% from min and 10% from max) of exon RPKM values for each sample.

### Diagnosis prediction model of PCS

Partek Genomics Suite’s support vector machine (SVM) classifiers with shrinking centroid was used to train models for predicting diagnosis. Models with a normalized correct rate greater than 85% were then tested on an independent validation data set to determine actual accuracy, sensitivity, and specificity.

### Statistics

Patient numbers were based on power analysis of previous data [9]. Graphpad Prism was used to perform normality, t-test, chi square, and Fisher’s exact test. A p-value <0.05 was considered significant. Analysis of variance (ANOVA) was performed on TMM normalized RPKM values to determine differential expression using Partek Genomics Suite at a significance cutoff of p <0.001. Principle component analysis and hierarchical clustering were used to group the data. Accuracy, sensitivity, specificity, and area under the curve (AUC) for the prediction models were also determined using Partek. Odds ratios were calculated using GraphPad Prism v 6.0.

## Supporting information

S1 DATA This is a zipped text file of exon read values for the sequencing samples. (GZ)

S2 DATA This is a zipped file of exon RPKM values for the sequencing samples. (GZ)

S3 DATA This is a zipped file gene aligned read values for the sequencing samples. (GZ)

S1 Table This is a text data file of the patient information for the sequencing data files. (TXT)
